# The Unseen Wounds: Why the Middle East’s Mental Health Catastrophe is a Regional Emergency

**DOI:** 10.34172/hpp.45874

**Published:** 2026-06-06

**Authors:** Hamid Allahverdipour

**Affiliations:** ^1^Editor-in-Chief, Health Promotion Perspectives; ^2^Department of Health Education & Promotion, Tabriz University of Medical Sciences, Tabriz 14711, Iran; ^3^Research Center of Psychiatry and Behavioral Sciences, Tabriz University of Medical Sciences, Tabriz, Iran

## Introduction

 International discourse concerning the Middle East has traditionally prioritised tangible crisis indicators, including mortality rates, destruction of infrastructure, and population displacement. Nevertheless, a less conspicuous but equally significant public health crisis is emerging across the region. Accumulating evidence suggests that tens of millions of people are enduring prolonged, transgenerational psychological trauma. Concurrently, mental health services remain profoundly underfunded, fragmented, and inaccessible owing to stigma, economic deterioration, and institutional neglect.^1,2^ This phenomenon encompasses several critical dimensions.

## Psychological Trauma and Mental Disorders

 Millions of children across the Middle East have been exposed to armed conflict and forced displacement. In Syria alone, the United Nations Children’s Fund (UNICEF) delivered mental health and psychosocial support to more than 50,000 children between January and August 2025, though this likely represents a small fraction of those in need.^3^ Among displaced Syrians and Iraqis residing in camp settings, 98.5% experienced at least one traumatic event, 86.3% faced three or more, and the prevalence of probable post-traumatic stress disorder (PTSD) and major depressive disorder approached 60%.^2^ In active war zones such as Gaza, adult PTSD prevalence ranges from 30% to 50%, while children face significantly elevated risks of PTSD, anxiety disorders, depression, and chronic physical illness, with consequences likely to persist across successive generations.¹ A 2025 survey conducted in Lebanon found that 47% of respondents met criteria for probable depression, 45% for anxiety, and 63% screened positive for any mental disorder. Furthermore, 55% of suicide decedents in Lebanon in 2024 were aged 38 years or younger, highlighting the particular vulnerability of young adults.^4^

## Existing Stigma and Inefficiency of the Health System

 Mental illness remains heavily stigmatised across many Middle Eastern societies, frequently attributed to spiritual frailty, familial dishonour, or supernatural forces (e.g., the “Evil Eye”) rather than recognised as a biomedical condition. Such perceptions delay help-seeking behaviour, diminish treatment adherence, and intensify social isolation.^5-6^ The region’s mental health infrastructure is critically insufficient. In Yemen, after a decade of armed conflict, only 40 psychiatrists are available to serve a population exceeding 30 million, yielding a ratio of one psychiatrist per 700,000 individuals.^7^ Across the Gulf Cooperation Council (GCC) states, an estimated investment shortfall of US$4.3 billion is required to provide 16,301 additional psychiatric beds. By comparison, the United Kingdom maintains 36.9 psychiatric beds per 100,000 population, whereas Saudi Arabia and the United Arab Emirates possess 14.0 and 18.4 beds per 100,000, respectively.^8^ Economic collapse has disrupted pharmaceutical supply chains; in Lebanon, shortages of the antipsychotic medication paliperidone at major psychiatric hospitals have resulted in relapse and repeated hospitalisation among patients with schizophrenia and bipolar disorder.^9^ Untreated mental disorders exert cascading effects on individuals, families, and broader society. Evidence associates war-related trauma with increased risks of domestic violence, substance use disorders, and community violence.¹ Moreover, childhood trauma is a well-established risk factor for lifelong psychiatric morbidity and chronic physical disease, thereby perpetuating cycles of disadvantage across generations.

## Regional Country Policies and Funding Gaps

 Global financing for mental health remains critically inadequate. According to the World Health Organization’s Mental Health Atlas 2024, median government expenditure on mental health has stagnated at 2% of total health budgets since 2017. Low-income countries spend as little as US$0.04 per capita annually on mental health, compared with US$65 per capita in high-income nations.^10^ The WHO Special Initiative for Mental Health has achieved incremental progress; however, persistent challenges in financing, workforce development, and service delivery remain.^11^

## Recommendations

 An urgent, evidence-based response is necessary, centred on three key strategies. First, to reduce stigma and foster community involvement, culturally tailored anti-stigma campaigns should be embedded in schools, primary healthcare clinics, and religious settings. Training faith leaders to identify clinical mental disorders and guide appropriate referrals represents a promising, low-cost approach.⁶ Second, in light of the severe shortage of mental health specialists, task shifting and capacity building are essential: non-specialist health workers, teachers, and community volunteers should receive structured training in psychological first aid and evidence-based psychosocial support. Third, ring-fenced financing for mental health is critical—both donors and national governments must dedicate a specific portion of health and humanitarian budgets to mental health, ensuring that for every dollar spent on physical health infrastructure, a meaningful share is directed toward psychiatric care and psychosocial programmes. Additionally, researchers should prioritise longitudinal studies on transgenerational trauma among war-affected populations to identify protective factors against chronic psychological distress. Furthermore, health organisations need to establish regional mental health surveillance with routine screening in primary care and train non-specialist providers by 2030 using the WHO mhGAP guidelines.

## Conclusion

 The mental health catastrophe in the Middle East is not a secondary consequence of regional instability—it constitutes a primary public health emergency with profound intergenerational implications. Without urgent investment in culturally appropriate, accessible, and adequately funded mental health services, the region’s capacity for social recovery and peacebuilding will remain severely compromised. As the evidence clearly demonstrates, there is no health without mental health, and no durable peace without psychological healing.
